# AMPK signaling is dysregulated in human tendinopathy and loss of AMPKα1 leads to cell, matrix and mechanical dysfunction in mouse Achilles tendon

**DOI:** 10.1101/2025.01.31.635920

**Published:** 2025-02-01

**Authors:** LeeAnn A. Hold, Nicole Migotsky, Syeda N. Lamia, Steph S. Steltzer, Sydney Grossman, Jessica Chen, Seung-Ho Bae, Paige Cordts, Tessa Phillips, Matthew J. O’Meara, Carol Davis, Susan V. Brooks, Moeed Akbar, Neal Millar, Megan L. Killian, Adam C. Abraham

**Affiliations:** 1 Molecular and Integrative Physiology, University of Michigan, Ann Arbor, MI, United States; 2 Orthopedic Surgery, University of Michigan, Ann Arbor, MI, United States; 3 University of Toledo College of Medicine, Toledo, Ohio, United States; 4 Gilbert S. Omenn Department of Computational Medicine and Bioinformatics, University of Michigan, Ann Arbor, MI, United States; 5 Medicinal Chemistry, University of Michigan, Ann Arbor, MI, United States; 6 Biomedical Engineering, University of Michigan, Ann Arbor, MI, United States; 7 Institute of Infection, Immunity and Inflammation, University of Glasgow, Glasgow, UK; 8 Lead contact

**Keywords:** Extracellular matrix, AMPKa1, Prkaa1, senescence, biomechanics, exercise

## Abstract

Tendinopathy is a tendon disorder that is caused by the failure to self-repair and has many pathological characteristics such as disorganized ECM and decreased cell viability. We have identified a possible target to combat these changes, AMPK, an energy stress sensor that was shown to maintain intracellular homeostasis. Through bulk RNA-sequencing of healthy and tendinopathic tendons from humans we have identified a novel finding of downregulation of AMPK signaling in the tendinopathic samples which suggests AMPK plays a role in tendon homeostasis. Our studies utilizing a conditional knock-out of Prkaa1 in tendon in mice showed that loss of AMPK results in degenerative ECM, impaired biomechanical properties and increased cellular senescence in Achilles tendon through the lifespan. Additionally, we found that exercise can delay senescence onset independent of AMPK. These findings highlight the importance of energy metabolism in tendon health which will assist in understanding the onset and progression of tendinopathy.

## INTRODUCTION

Tendons are poorly vascularized tissues rich in extracellular matrix (ECM) that transmit force from muscle to bone. The mechanical demand placed on these tissues requires a precise balance of metabolic processes to maintain functional homeostasis^1,2^. Tendinopathy, is a tendon disorder and while the precise causes and characteristics remain to be elucidated, it is associated with results in pain, swelling, and diminished biomechanical properties. is a clinical problem that affects ~3.5 million people in the US^3^. Risk factors for tendinopathy include age, metabolic disorders and limited exercise^3^. Tendinopathy is caused by failure of tendon to self-repair and is characterized by degenerative ECM, decreased cell viability, and poor biomechanical function^4^. Currently, treatment for tendinopathy has limited non-surgical treatment options^3^, therefore elucidating targets for a druggable therapy will improve current clinical limitations. Previously, Wu et. al established a promising molecular mechanism for treatment of diabetic induced tendinopathy through the AMP-activated protein kinase (AMPK) pathway^5^. The AMPK pathway is an intracellular stress response activated in times of nutrient deficiency, and exercise to regulate energy balance and maintain homeostasis^6^. In response to a decrease in the ATP:AMP ratio, AMPK is phosphorylated and redirects metabolism to stimulate catabolic processes while simultaneously decreasing anabolic processes^6^. While AMPK is a critical regulator of homeostasis in other tissues^7,8^ as well as a potential regulator of ECM remodeling in musculoskeletal disease^9^, little is known about how AMPK regulates energy balance and homeostasis in tendon. A key pathological characteristic of tendinopathy includes dysregulation of homeostasis, such as matrix disorganization and decreased cell viability^3^. Additionally, regulation of metabolic mechanisms play a role in tendon healing and degeneration^10,11^ establishing the potential for metabolic modulators as therapeutic targets for tendinopathy. Therefore, the potential in targeting AMPK motivates the need to define the role of AMPK in tendon homeostasis.

The basal metabolic rate of tendon is relatively low compared to other musculoskeletal tissues, possibly related to the high number of quiescent cells present in tendon during homeostasis^12^. The quiescent phenotype presents during times of limited nutrients and is coupled with a reversable cell cycle arrest. It is believed that tendon fibroblasts (TFs) become predominantly quiescent in the early stages of maturation^13^. Grinstein et. al showed in mice that after post-natal day 30 mitotic activity declines significantly and few cells continue to divide through the remainder of life^13^. Regulation of quiescence through AMPK has been shown to be age dependent in muscle stem cells^14,15^.With age, AMPK activity is dampened resulting in a push towards senescence through nuclear translocation of cyclin inhibitors^14^. Furthermore, activation of AMPK restored cell survival, through upregulation of autophagy and reduced nuclear translocation of cyclin inhibitors, in aged skeletal muscle stem cells^14^. These trends may be consistent in tendon, and in a study by Dai et. al, it was shown that that activation of the AMPK signaling cascade through metformin reduced the effects of aging *in vivo* and *in vitro*^16^. Using tendon progenitor cells *in vitro*, metformin reduced senescence markers p16 and p21^16^. Additionally, they showed metformin reduced the aging phenotype by inhibiting senescence marker p16 and the formation of heterotopic ossification in 10 month-old rats^16^. Taken together, these data implicate AMPK as a possible target to regulate cellular fate in tendon and prevent age-associated functional decline.

AMPK is a heterotrimeric complex composed of α, β and γ subunits^6^. There are two α subunits, α1 and α2: AMPKα1 is ubiquitously expressed in all tissues while AMPKα2 is dominant in skeletal muscle, heart, and liver^17,18^. Reduction of AMPKα1 has been associated with musculoskeletal diseases such as osteoarthritis (OA)^19^. For example, loss of AMPKα1 leads to accelerated progression and increased severity of OA^9,19^. However, while dysregulation of AMPKα1 in some degenerative musculoskeletal diseases is known, the mechanisms that underlie its role in tendinopathy remain unclear.

In this study, we identified that the AMPK signaling pathway is transcriptionally suppressed in patients with tendinopathy. Additionally, we found that AMPK is necessary for tendon homeostasis by mediating expression of *Prkaa1* in TFs. We used Cre-lox to target deletion of *Prkaa1*, the gene that encodes AMPKα1, in tendon progenitors in mice. This targeted deletion led to normal development of tendons; however, we saw dysregulation of cell cycle and ECM biological processes in the transcriptome at 1 month of age (1M). By 3 months of age (3M), we found that tendons lacking *Prkaa1* had impaired mechanical properties and upregulation of senescence associated marker, p21. With increased age, tendons lacking *Prkaa1* developed expansive ectopic calcification and had elevated markers of senescence associated p16 and p21. Voluntary wheel running was beneficial in tendons of *Prkaa1*-deficient mice and led to increased ECM organization and suppressed p16 and p21. Our results demonstrate AMPKα1 is necessary for tendon homeostasis and exercise, an energy demanding process, which can delay senescence onset independent of AMPK α1 suggesting energy balance is crucial for cellular viability in tendon.

## RESULTS

### Metabolic pathways and AMPK signaling are dysregulated in tendinopathy

**Figure 1:**
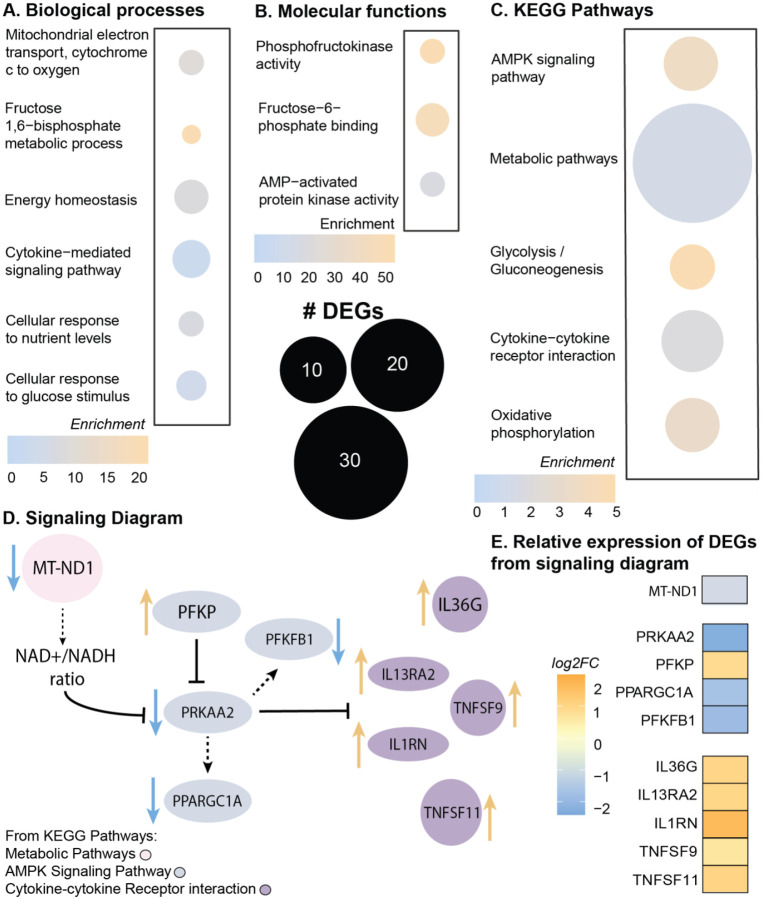
Metabolic pathways and AMPK signaling are dysregulated in human tendinopathy. (A-C) Enrichment quantification of differentially expressed genes (DEGs) from bulk RNA sequencing of human tendinopathic samples compared to healthy control tendons. (A. Enriched GO biological processes; B. Enriched GO molecular functions; C. Enriched Kegg pathways.) (D) Signaling diagram from individual DEGs from KEGG pathways (Gray: DEGs in AMPK signaling pathway; Pink: DEGs from metabolic pathways; Purple: DEGs in cytokine-cytokine receptor interaction pathway; blue arrow representative of downregulated DEGs, orange arrow representative of upregulated DEGs) (E) Relative expression of DEGs from signaling diagram (log2 fold change) from human tendinopathic samples compared to healthy control tendons. n= 7 samples/group. Adjusted p-value: p<0.05, log2FC±1.5

Using bulk RNA-sequencing, we compared healthy hamstring tendon to diseased rotator cuff tendons from human donors and found a downregulation of multiple genes associated with the mitochondrial electron transport chain and energy production, suggesting an overall dysregulation in metabolism (Fig. 1, Table S1 for patient demographics). Enriched biological processes were related to energy homeostasis, response to nutrients, and cytokine signaling (Fig. 1A). Additionally, enriched molecular functions (Fig 1B) and KEGG pathways were also related to AMPK activity and signaling (Fig. 1C), respectively, with KEGG pathways also enriched for regulation of energy metabolism (i.e. glycolysis and oxidative phosphorylation), metabolic pathways and cytokine signaling (Fig. 1C). When analyzing the differentially expressed genes (DEGs) produced from the KEGG pathway results, the AMPK signaling pathway was primarily driven by six DEGs, of which 5 were downregulated in tendinopathic tendons (Fig. 1D,E), with phosphofructokinase (*PFKP*) as the only upregulated gene (Fig. 1D,E). The top 10 hits for DEGs associated with metabolic pathways were also downregulated with tendinopathy, including genes associated with the mitochondrial electron transport chain (Fig. 1D,E). Specifically, tendinopathy samples had downregulated mitochondrial encoded NADH dehydrogenase 1 (*MT-ND1*; Fig. 1D,E), which regulates mitochondrial complex one and the NAD^+^/NADH ratio^20^. A decrease in the NAD^+^/NADH ratio has been associated with decreased AMPK activation and increased production of the senescence associated secretory phenotype (SASP)^21^. Additionally, DEGs associated with the cytokine-cytokine receptor interaction pathway included activation of multiple interleukins (e.g. *IL1*, *IL13*, and *IL36*; Fig. 1D,E), another characteristic of the SASP^22^. Considering these findings, we sought to identify the role on AMPK in the mouse Achilles tendon in an age-dependent manner.

### Prkaa1^Scx-Cre^ cKO mouse tendons showed dysregulation of cell cycle processes, increased calcification and impaired biomechanical properties.

**Figure 2:**
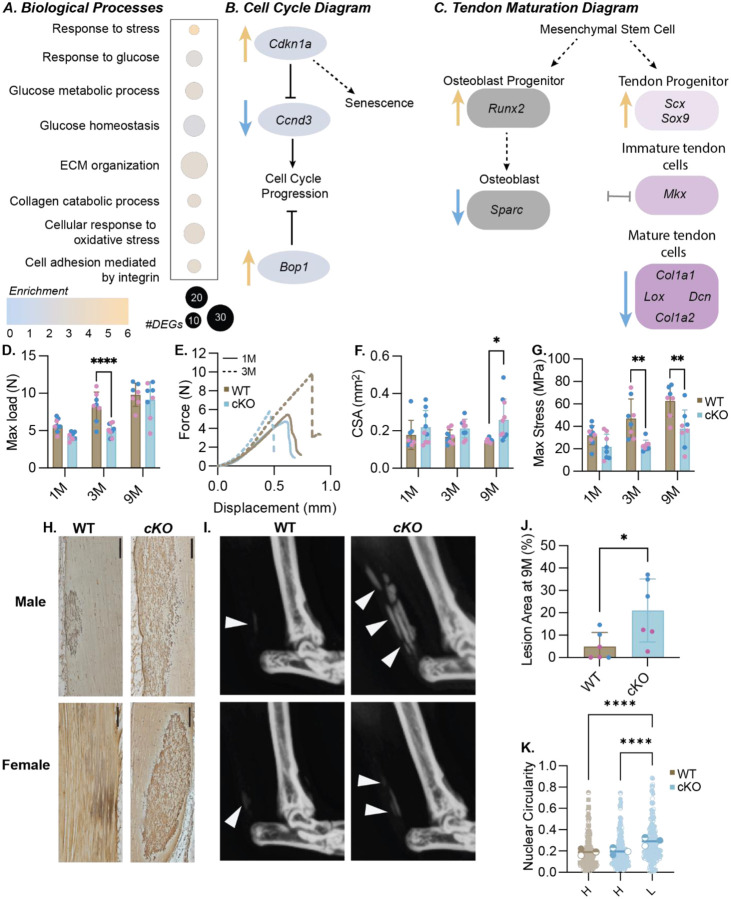
Phenotypic characterization of Prkaa1^Scx-Cre^ cKO Achilles tendons. (A) Quantification of GO biological processes found during bulk RNA sequencing in 1M female Prkaa1^Scx-Cre^ cKO mice compared to WT controls. (B) Signaling diagram created from cell cycle related DEGs in Prkaa1^Scx-Cre^ cKO mice compared WT controls. (blue arrow representative of downregulated DEGs, orange arrow representative of upregulated DEGs) (C) Signaling diagram created from tendon maturation related DEGs in Prkaa1^Scx-Cre^ cKO mice compared WT controls. (blue arrow representative of downregulated DEGs, orange arrow representative of upregulated DEGs, gray bay representative of no significant change) n=3 samples/group. Adjusted p-value: Adjusted p-value: p<.05, log2FC±1.5 (D-G) Biomechanical outcomes from 1, 3 and 9M WT and Prkaa1^Scx-Cre^ cKO Achilles tendons (D. Maximum Load before failure; E. Representative Force vs Displacement curve for 1M and 3M WT and Prkaa1^Scx-Cre^ cKO Achilles tendons F. Cross-sectional area (CSA); G. Maximum stress). n =6 – 10/group, * p<.05, ** p<0.01; two-way ANOVA with Sidak correction. (H) Representative X-ray images of dystrophic calcification, highlighted by white arrows in the 9M WT and cKO Achilles tendons. (I) Representative silver nitrate-stained images in 9M WT and Prkaa1^Scx-Cre^ cKO Achilles tendons. Scale bar = 200μ m. (J) Quantification of positive silver nitrate-stained lesions in 9M WT and Prkaa1^Scx-Cre^ cKO Achilles tendons. n =6/group, * p<0.05. (K) Quantification of nuclear circularity 9M WT and Prkaa1^Scx-Cre^ cKO Achilles tendons. ((H) = healthy, (L) = lesion; n =3/group, ****p<.0001; by paired t-test.

In the Achilles tendons of mice at 1M of age, we identified 621 upregulated and 1008 downregulated genes in Prkaa1^Scx-Cre^ cKO females compared to age-matched WT females using bulk RNA sequencing, yet transcriptional differences were not observed in male mice at 1M or 3M of age. Prkaa1^Scx-Cre^ cKO tendons from female mice at 1M had enriched biological processes related to cell cycle regulation, matrix organization and response to nutrients compared to WT females (Fig. 2A). When comparing cell cycle DEGs, we found a robust upregulation of cell cycle arrest markers in tendons from female Prkaa1^Scx-Cre^ cKO mice such as *Ccngd3*, the gene that encodes cyclin D3, and *Cdkn1a*, the gene that encodes p21 (Fig. 2B). Finally, when comparing metabolism DEGs we identified a significant upregulation of *Bop1*, a known inhibitor of cell cycle progression, in cKO tendons compared to WT at 1M^23^ (Fig.2B). Cellular senescence is defined as terminal growth arrest in cells even if stimulated by mitogens, however they are metabolically and synthetically active^24^. Although there is no clear pathway to senescence, early markers of senescence include changes in glucose metabolism and increased cyclin kinase activity. These findings suggest that Achilles tendons in Prkaa1^Scx-Cre^ cKO mice exhibit a transcriptional profile that supports the onset of senescence^25,26^. Senescent cells are also known to contribute to ECM remodeling^27^. When comparing tendon and matrix related DEGs, we found *Scx* was significantly upregulated in female Prkaa1^Scx-Cre^ cKO tendons, while *Lox*, *Loxl3* and both *Col1a1* and *Col1a2* were downregulated compared to WT female tendons (Fig. 2C). A decrease in *Scx* expression in tendon indicates a shift towards a more mature tendon state^28^. Our results showing an increase in Scx mRNA expression in the cKO cells suggest while the WT cells are transitioning into a more mature tendon the cKO cells transcriptionally resemble a progenitor tendon cell. At the protein level, LOX catalyzes the formation of crosslinks in collagen and is essential for the formation of collagen fibril, decreased *Lox* gene expression has been associated shown to reduce the ability to withstand load in *in vitro* tendon constructs^29^. We found the Prkaa1^Scx-Cre^ cKO Achilles tendons had impaired mechanical function compared to WT controls.

First, we found the maximum failure load of Prkaa1^Scx-Cre^ cKO Achilles tendons trended downward at 1M (p = 0.0509) and was significantly reduced at 3M compared to WT controls (Fig. 2D,E), however the maximum load equilibrated by 9M of age (Fig. 2D). Prkaa1^Scx-Cre^ cKO Achilles tendons were significantly larger (cross-sectional area, CSA) compared to WT controls at 9M (Fig. 2F). When normalizing the load to CSA we found peak mechanical stress of Prkaa1^Scx-Cre^ cKO Achilles tendons was also significantly reduced at 3M and 9M of age compared to WT controls (Fig. 2G). When comparing at 3% stain the tangent modulus of Prkaa1^Scx-Cre^ cKO Achilles tendons were significantly reduced at 3M of age (Table S2). Finally, we saw no changes to stiffness between WT and cKO tendons at any age we investigated (Table S2). The functional changes we observed were likely not due to impaired muscle function, as we saw no differences in muscle force production in Prkaa1^Scx-Cre^ cKO mice at 3M (Tables S3–9). We next analyzed the Prkaa1^Scx-Cre^ cKO Achilles tendons histologically and identified positive silver nitrate-stained areas we will report as lesions (Fig.2H), Quantification of silver nitrate-stained lesions at 9M of age showed that Prkaa1^Scx-Cre^ cKO Achilles tendons had a significantly greater percent of positive lesion area compared to WT controls (Fig. 2J) and X-ray confirmed these regions appeared as radio-opaque ectopic calcification (Fig. 2I). Cells contributing to ectopic calcification can exhibit nuclear swelling due to increased stress caused by aberrant calcium levels^30^. In line with this, we found the cell nuclei in lesions at 9M were rounder compared to non-lesion “healthy” tendon within Prkaa1^Scx-Cre^ cKO Achilles tendons as well as in WT tendons (Fig. 2K). Finally, we found that collagen organization, measured using quantitative polarized light imaging (qPLI) of fiber birefringence on histological sections, was not different between Prkaa1^Scx-Cre^ cKO tendons and WT controls at 1, 3 and 9 months of age (Fig. S1A,B). In summary we identified dysregulation of matrix interaction signaling as well as markers of pre-mature senescence therefore, we sought to identify how loss of *Prkaa1* effects ECM specific adhesion and if Prkaa1^Scx-Cre^ cKO tendons exhibited additional senescence markers with age.

### Prkaa1^Scx-Cre^ cKO Achilles tendon fibroblasts have altered ECM specific adhesion and show an increase in senescence associated markers.

**Figure 3:**
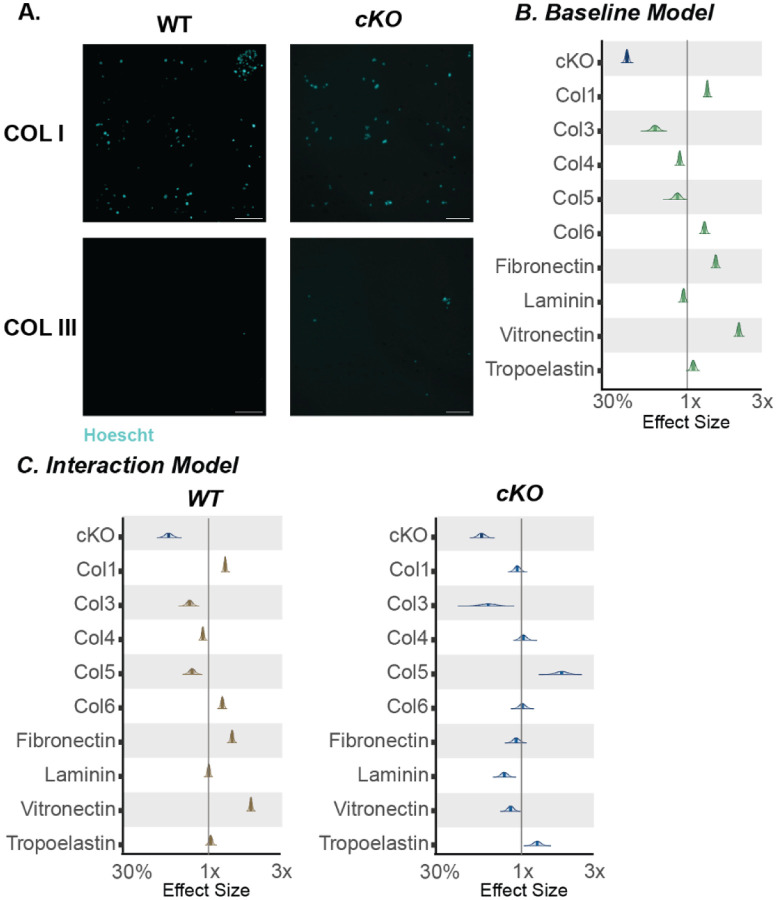
Tendon fibroblasts from Prkaa1^Scx-Cre^ cKO mice have impaired ECM specific adhesion. (A) Representative images of Hoechst staining used for COLI and COL III. (B-C) Posterior distributions with median and 80% confidence intervals of Bayesian regression models quantified from nuclear counts (B. Baseline; C. Interaction; Grey bar represents median number of all nuclear counts regardless of genotype) n = 3/group.

**Figure 4:**
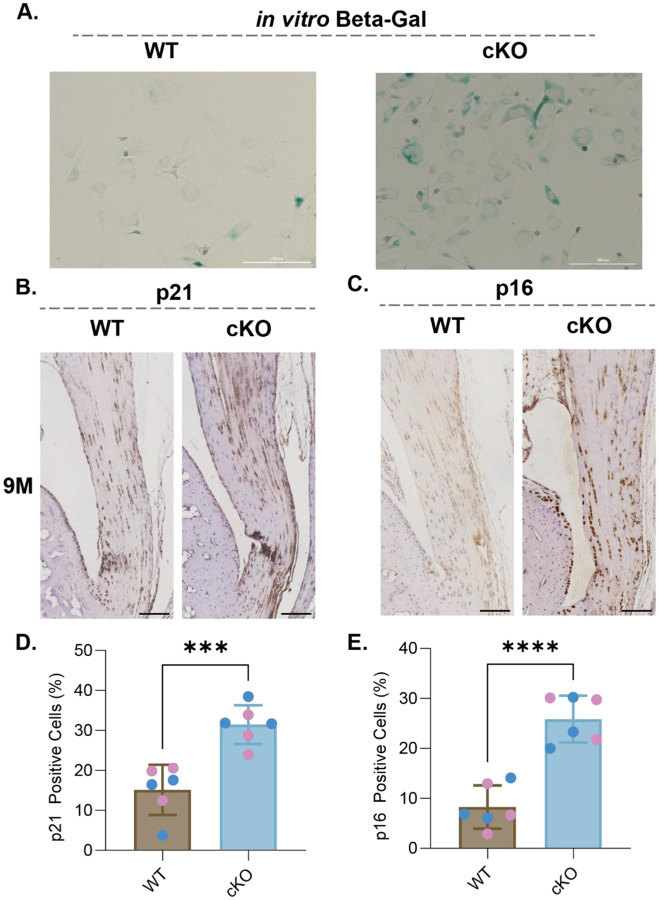
Prkaa1Scx-Cre cKO tendon fibroblasts had increased expression of senescence markers in vitro and in vivo. (A) Representative Beta-Galactosidase stain of WT and Prkaa1^Scx-Cre^ cKO tail TFs *in vitro* after five days in culture. Scale bar = 200μm. n=3/group. (B and C) Representative IHC images in 9M WT and Prkaa1^Scx-Cre^ cKO Achilles tendons of (B) p21 and (C) p16 staining using DAB. (D and E) Quantification of (D) positive p21 cells and (E) positive p16 cells in 9M WT and Prkaa1^Scx-Cre^ cKO Achilles tendons. Scale bar = 500μ m. n =6/group (pink = female; blue = male), ***p<0.001, ****p<0.0001; two-way ANOVA with Sidak correction.

To compare cellular adhesion between Prkaa1^Scx-Cre^ cKO and WT TFs we plated cells on an array of 36 matrix spots comprised of individual and mixtures of 9 ECM component proteins and measured nuclear counts after 24h as a proxy for adhesion. Across all conditions, spot counts ranged from 0 to 408 with a median of 95. To analyze the drivers of this variation, we fit a Bayesian general linear regression models with genotype and matrix proteins as covariates and using weakly informative priors and compared the posterior distributions over regression weights. As a baseline model, we assumed no statistical interaction between genotype and the matrix proteins and since the outcomes are counts, we used a Poisson link function. We found that Prkaa1^Scx-Cre^ cKO TFs were less adherent than WT TFs (Fig. 3A). Additionally, collagen (COL) 1, COL6, fibronectin and vitronectin were more permissive while COL3 was less permissive for adhesion regardless of genotype (Fig. 3A). Considering an interaction model where genotype and matrix interact suggested there was a preference of Prkaa1^Scx-Cre^ cKO TF adhesion for Col5 and tropoelastin. Additionally, Prkaa1^Scx-Cre^ cKO TFs showed a decreased preference for COL1 and laminin (Fig. 3B). There is increasing evidence showing the composition of the ECM potentiates cellular senescence^31^. We identified senescence in Prkaa1^Scx-Cre^ cKO tail TFs using Beta-Galactosidase staining (Fig. 4A). Two cell-cycle inhibitors that are commonly regarded as markers of senescent cells are the cyclin-dependent kinase inhibitors p21 and p16^32^. Here, we used immunohistochemistry of the Achilles tendon and identified a significant increase in p21 and p16 in Prkaa1^Scx-Cre^ cKO mice compared to WT controls at 9M of age (Fig. 4B–E). It is important to note that even in the 9M WT samples we saw >10% of the tendon cell population expressed both p16/p21. Cells expressing senescent markers can play critical roles in tissue regeneration and homeostasis^33^, suggesting that while we saw a significant increase in senescent markers in Prkaa1^Scx-Cre^ cKO Achilles tendons, senescence may be a normal process in tendon homeostasis. Moreover, given the ability to remove senescent cells through exercise, we decided to use voluntary wheel running to determine the effects of exercise on senescence in the Achilles tendon independent of AMPK. However, given our cells ability to respond to nutrient depletion is impaired we were unsure how these tendons would respond to exercise. Therefore, we gave WT and Prkaa1^Scx-Cre^ cKO access to voluntary running wheels.

### Exercise increases organization and reduces the onset of senescence in Prkaa1^Scx-Cre^ cKO Achilles tendons.

**Figure 5:**
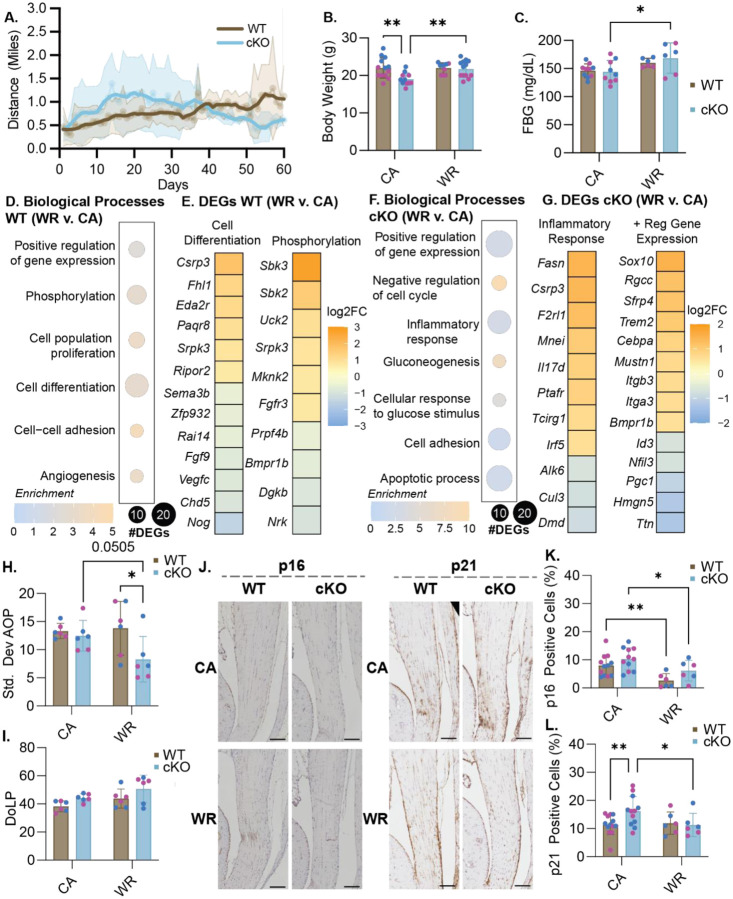
Effects of exercise on Prkaa1^Scx-Cre^ cKO mice. (A) Quantification of distance run for WT and Prkaa1^Scx-Cre^ cKO mice. (B) Quantification of body weight for WT and Prkaa1^Scx-Cre^ cKO WR and CA mice. (C) Quantification of fasting blood glucose (FBG) mg/dL for wheel running groups for WT and Prkaa1^Scx-Cre^ cKO WR and CA mice. n =6–12/group, females = pink; males = blue. * p<0.05, ** p<0.01; two-way ANOVA with Sidak correction. (D) Quantification of GO biological processes found during bulk RNA sequencing in WT WR mice compared to CA controls. (E) Quantification of the log2 fold change of DEGs from WT mice from biological processes WR mice compared to CA controls (Cell differentiation; Phosphorylation). (F) Quantification of GO biological processes found during bulk RNA sequencing in Prkaa1^Scx-Cre^ cKO mice WR mice compared to CA controls. (G) Quantification of the log2 fold change of DEGs in WR Prkaa1^Scx-Cre^ cKO mice from biological processes (Inflammatory response; Positive regulation of gene expression). n= 3–6 samples/group. Adjusted p-value: Adjusted p-value: p<.05, log2FC±1.5 (H and I) Quantification of alignment for WT and Prkaa1^Scx-Cre^ cKO WR and CA mice (H. Degree of linear polarization (DoLP); I. Standard Deviation Angle of Polarization (AOP)), females = pink; males = blue (J) Representative IHC images of p16 and p21 in CA and WR Prkaa1^Scx-Cre^ cKO Achilles tendons. Scale bar = 500μ m.Quantification of p16 positive cells. Quantification of positive p21 cells. n =6/group, * p<0.05, ** p<0.001, ***p<0.0001; two-way ANOVA with Sidak correction. (K) Quantification of positive p16 cells. n =6/group, females = pink; males = blue. * p<0.05, ** p<0.01, ***p<0.001; two-way ANOVA with Sidak correction. (L) Quantification of positive p21 cells. n =6/group, females = pink; males = blue. * p<0.05, ** p<0.01, ***p<0.001; two-way ANOVA with Sidak correction.

We identified a significant linear relationship between distance ran and days with wheels in the WT mice (r^2^=0.7295) during voluntary wheel running (WR) but not the Prkaa1^Scx-Cre^ cKO mice (r^2^=0.02202) showing the WT mice ability to adapt (Fig. 5A). The body weight of Prkaa1^Scx-Cre^ cKO mice was significantly lower than WT mice in cage activity (CA) group but was rescued with WR (Fig. 5B). Additionally, fasting blood glucose (FBG) was significantly elevated in the Prkaa1^Scx-Cre^ cKO WR mice compared to CA mice at 3M of age (Fig. 5C). Voluntary wheel running can influence the metabolic activity in rats where *Gapdh* was shown to be significantly downregulated as well as an increase in overall food intake compared to controls^34^. Using bulk RNA sequencing of Achilles tendons, we found 109 upregulated and 78 downregulated genes with WR compared to CA in WT tendons. In WT tendons, enriched biological processes were related to protein phosphorylation, cell differentiation, and positive regulation of gene expression and angiogenesis (Fig. 5D). When comparing DEGs from cell differentiation processes, we found upregulation of *Csrp3* (Fig. 5E). Additionally, when comparing DEGs related to phosphorylation, we saw upregulation of *Sbk3* and *Sbk2* and downregulation of *Bmpr1b*. Protein phosphorylation, the most common post-translational modification, utilizes ATP and is representative of good energy balance within a cell^35^ suggesting voluntary wheel running promotes energy homeostasis in tendons. In Prkaa1^Scx-Cre^ cKO tendons, we found 169 upregulated and 272 downregulated genes with WR compared to CA. Enriched biological processes included positive regulation of gene expression, inflammatory response, and apoptosis (Fig. 5F). When comparing DEGs from inflammatory response we again found upregulation of *Csrp3* as well as *Cul3* (Fig.5G). When looking at the positive regulation of gene expression we found upregulation of *Itgb3*, *Itga3* and *Bmpr1b* (Fig.5G). Integrin expression is upregulated with increased mechanical signaling, thought to be necessary to transmit mechanical stimuli to the cells during loading^36^. When looking at the structure of the tendon we found a significant increase in tendon organization in the WR Prkaa1^Scx-Cre^ cKO mice when comparing to the WT WR and a trend upward when comparing to the Prkaa1^Scx-Cre^ cKO CA group (p = 0.0505; Fig. 5H) However, we found no significant change in tendon alignment between the groups (Fig. 5I). Immunohistochemistry of Achilles tendons revealed a significant decrease in senescence-associated p21 with WR compared to CA in Prkaa1^Scx-Cre^ cKO mice (Fig. 5J). Finally, we found WR significantly suppressed senescence-associated p16 regardless of genotype (Fig. 5J).

## DISCUSSION

Defining the role of energy metabolism in the context of tendon health and disease is critical for the advancement of non-surgical therapeutics for patients with tendinopathy. While the role AMPK has been established as a regulator of ECM organization and homeostasis in other musculoskeletal tissues^7–9,19^, the role of AMPK in tendon homeostasis and tendinopathy has previously been unclear. Here we have demonstrated that AMPK signaling is dysregulated in human tendinopathy patients and *Prkaa1* is necessary to maintain homeostasis in adult mouse Achilles tendon. These data demonstrated that loss of *Prkaa1* resulted in premature senescence as well as accelerated onset of ectopic calcification. Additionally, we found that exercise delays the onset of senescence in a *Prkaa1*-independent manner suggesting energy usage is essential for the maintenance of tendon health.

To the best of our knowledge, we are the first to identify dysregulation of the AMPK signaling pathway in tendinopathy patients. We identified upregulation of genes related to SASP production in tendinopathy patients and these trends are consistent when evaluating other data sets from tendinopathic patients^37^. In a study by Zhu et. al, screening for biomarkers of tendinopathy revealed common processes dysregulated in tendinopathy were related to the cell cycle, mitochondria function and pro-inflammatory responses as well as mTOR and NF-κB signaling pathways^37^. Additionally, other musculoskeletal diseases can be driven by senescence and the SASP^38–40^. In osteoarthritis, it is speculated that synovial macrophages, fibroblasts and osteoclasts promote SASP production, leading to an inflammatory response that enhances disease progression^39,40^. Taken together, our findings, along with previous insights into musculoskeletal disease, suggest progression of tendinopathic disease may be driven by the presence of senescent fibroblasts and maintained through an increased proinflammatory response.

We identified the formation of ectopic calcification in Prkaa1^Scx-Cre^ cKO Achilles tendons, especially males. Surprisingly, our bulk RNA sequencing results showed an upregulation of both *Runx2* and *Dlx5* in female, but not male, Prkaa1^Scx-Cre^ cKO tendons. The relationship between AMPK and RUNX2 can vary depending on cell type^41–43^. In osteoblasts, AMPK can regulate Runx2 post-transcriptionally and promote osteogenesis^41^. However, in vascular mesenchymal stem cells, AMPK can downregulate Runx2 expression, and loss of AMPKα1 can promote osteoblastic differentiation^43^. Spatially, we noticed the presence of calcification near the peritenon, suggesting the possibility of cell migration into the tendon core before the onset of calcification. Peritenon cells have been identified as being more stem-like than tendon core cells, expressing several stem cell markers^44^. Additionally, peritenon cells are capable of undergoing osteogenic differentiation *in vitro*^44^. Taken together, TFs and peritenon cells may respond similarly to mesenchymal stem cells with loss of AMPK, pushing them toward an osteogenic state. Future directions will explore the role of the peritenon in the formation of ectopic calcification and how migration and trans-differentiation of these cells may be induced by SASP. The formation of calcification within soft tissues has been linked to increase of senescent cells^16,45,46^. Here we identified loss of *Prkaa1* results in both premature senescence and ectopic calcification in the mouse Achilles tendon. Our data supports proposed mechanisms in which an increase p21 responds to a replicative related process (e.g DNA damage) which precedes the increase in p16, while both together correspond to late senescent cells and the presence of CDK inhibition and cell cycle arrest^47^. Our data also supports previous findings in tendon suggesting that modulation of the AMPK/mTOR signaling cascade via metformin delays the accumulation of senescent cells, cell morphological changes, and calcification^16,48^. Surprisingly, we found that while we had no sex differences in our senescence associated marker expression, we had distinct differences in calcification area between our male and female mice. Future directions will explore estrogen as a protector of calcification in the female tendon as well as identify the potential of the SASP to induce calcification and additional therapeutics to delay onset of senescence.

Integrins regulate the onset of senescence in fibroblasts^49^ AMPK is a known regulator of β−1 integrin^50^. Consequently, loss of AMPK in fibroblasts can increase cell spreading as well as fibronectin fiber formation^50^. Here, we found transcriptional changes of multiple integrins in both the 1M timepoint as well as with exercise in Prkaa1^Scx-Cre^ cKO tendons. Additionally, we found that TFs from Prkaa1^Scx-Cre^ cKO tails have impaired ECM substrate-specific adhesion. Different ECM substrates have unique integrin binding properties, and taken together these data suggest that AMPK activity is responsive to different ECM substrates and integrin accessibility.

The reduction of senescent cells through exercise has been seen in fibrotic disease models^51,52^. Here we found that voluntary wheel running delayed the onset of senescence markers, p16 and p21, as well as increased cell cycle progression markers (i.e. Regulator of cell cycle progression (*Rgcc*)) in Prkaa1^Scx-Cre^ cKO tendons. In addition to regulating cell cycle progression, *Rgcc* may have protective effects against fibrosis, and stimulation of *Rgcc* correlated with enrichment of ECM organization pathways^53^. These trends correlate with our findings that exercise induced a significant increase in *Rgcc* gene expression as well as increased collagen alignment and integrin expression in Prkaa1^Scx-Cre^ cKO tendons.

In summary, these data show that AMPK, which is dysregulated in tendinopathy, is necessary for the maintenance of homeostasis and mechanical function in the mouse Achilles tendon. Additionally, we found exercise has a protective effect against senescence independent of AMPK and promotes energy homeostasis in tendon. These findings highlight the importance of energy balance metabolism in tendon health and overall improves our understanding of the onset and progression of tendinopathy as well as identification of potential treatment options for tendon disease.

### Limitations of the study

Although our data parallels other bulk-RNA sequencing of human Achilles tendinopathy, our cohort of patients was small with only seven per group focusing on the rotator cuff. Additionally, despite conducting bulk-RNA sequencing at 1M and 3M on female and male mice, we only observed large transcriptional differences in our female 1M mice. We speculate either our timepoint was too late to capture similar differences in the male mice or the phenotype was driven by post translational or other modifications. Future work with proteomics could assist in divulging these differences.

## RESOURCE AVAILABILITY

### Lead contact

Further information and requests for resources and reagents should be directed to and will be fulfilled by the lead contact, Adam Abraham (adaabrah@med.umich.edu).

### Materials availability

This study did not generate new unique reagents.

### Data and code availability

- Bulk-seq data have been deposited at GEO at GEO: accession number and are publicly available as of the date of publication.
- All raw data will be deposited to Deep Blue Data

## STAR★METHODS

### EXPERIMENTAL MODEL AND STUDY PARTICIPANT DETAILS

#### Human

Late-stage RC (supraspinatus) tendon samples and control hamstring (semitendinosus) tendon samples were provided by the School of Infection and Immunity at the University of Glasgow. Informed consent was obtained from participants, and the use of tissue for research was approved through the West of Scotland Research Ethics Service (REC 16/WS/0207).

#### Animal Models

All procedures were approved by the Institutional Animal Care and Use Committees at the University of Michigan. ScxCre; Prkaa1^fl/fl^ male mice were bred with Prkaa1^fl/fl^ female mice to generate Prkaa1^Scx-Cre^ (cKO). Prkaa1^fl/fl^ (WT) and Prkaa1^Scx-Cre^ cKO (cKO) mice were generated for evaluation of Achilles tendon structure and function at 1-, 3-, and 9-months (1M, 3M, 9M). All mice were kept in standard housing conditions with ad libitum access to water and chow throughout the experiment.

### METHOD DETAILS

#### RNA isolation and sequencing of human tendon

Tissues were collected and stored in RNALater, these were subsequently physically disrupted using a ball mill homogenizer. RNA extraction was performed using RNeasy Mini Fibrous Tissue Kit with on column deoxyribonuclease I treatment according to manufacturer’s instructions. RNA quality and quantity was obtained using Qubit fluorometer and the RNA Pico kit and Bioanalyzer 2100 system.

The NEBNext Ultra II RNA Library Preparation for Illumina Kit was used to prepare the samples for sequencing following the manufacturer protocol. Dual Index Primers were used in the NEXNext Ultra II RNA Library Preparation for Illumina protocols. Library qualities were confirmed using Qubit fluorometer and high sensitivity DNA kit and Bioanalyzer. Samples were then sequenced using an Illumina NextSeq500 Instrument (at University of Glasgow Polyomics Facility (Glasgow Polyomics, University of Glasgow).

Differential gene expression was determined from count matrices with a paired design (e.g. Healthy vs. Diseased) in DESeq2 in R/Bioconductor^54^.We used the Database for Annotation, Visualization, and Integrated Discovery (DAVID) to analyze biological processes and Kyoto Encyclopedia of Genes and Genomes (KEGG) pathways^55,56^.

#### Wheel Running

At 1M of age WT and Prkaa1^Scx-Cre^ cKO mice were randomly assigned to either remain at cage activity (CA) or were singly housed with voluntary access to running wheels (WR) for 8 weeks in standard mouse cages. Daily wheel rotations were collected and recorded. One week prior to euthanasia mice were fasted for 6hr to collect fasting blood glucose (FBG) measurements.

#### Biomechanical testing of Achilles tendon

At time of euthanasia, one hindlimb from each mouse was immediately frozen at −20°C. Simple randomization of samples prior to Achille’s tendon dissection was performed and tester was blinded to all experimental groups. 24 hours before the start of mechanical testing, limbs were thawed at 4°C and prepared for photogrammetry and biomechanical testing. Both the plantaris tendon and muscle were carefully removed and the Achilles tendons and calcaneus were left intact. Cross-sectional areas (CSA) of the Achilles tendon were measured using photogrammetry with a custom pinch clamp holder attached to a motor controller (Arduino, Ivrea, Italy). With the muscle and Achilles hanging free of the pinch clamp, at least 50 consecutive images of the pinch clamped tendon were acquired using a 12mm focal length lens (Basler Fujinon Lens, Ahrensburg, Germany). Using Metashape software (Agisoft, St. Petersburg, Russia), images were aligned and converted first to a sparse point then to a dense point cloud, to create an STL surface mesh of the tendon. CSA was defined as the smallest area from the STL surface mesh generated from the Achilles tendon and was measured using a slice analysis tool in Dragonfly (Comet Technologies Canada Inc., Montreal, Canada).

A custom 3D printed fixture secured the calcaneus into place (FormLabs 3B, Somerville, MA, USA), and the proximal Achilles tendon was clamped in a textured grip and screwed into place (Imada, Northbrook, IL, USA). The assembled grip with the secured Achilles tendon was placed into a phosphate buffered solution (PBS) bath at 37°C using a temperature controller (MA160, Biomomentum Inc., Laval, Quebec, Canada) and secured with a pin to a tensile testing frame with a multi-axis load cell (±70 N; Mach-1 VS500CST, Biomomentum, Laval, Quebec, Canada). Samples were preloaded to 0.1 N, and gauge length was measured as the distance between the secured calcaneus and the textured grip.

The Achilles tendons were then preconditioned for 10 cycles (±0.1 N at 0.1mm/sec) followed by the load to failure test at 0.1mm/sec. Off-axis load (forces in X and Y, torques in X, Y, Z) were collected to assess off-axis loading for the duration of the experiment. Instantaneous grip-to-grip strain was calculated as the displacement divided by the original gauge length, *L*_0_. For the 1M, 3M and 9M WT and cKO age-dependent tests maximum load, maximum stress, and maximum strain were calculated using a custom MATLAB script (R2017 or later, MathWorks, Natick, USA). Data between cKO and WT Achilles tendons at 1M, 3M and 9M were compared using a two-way ANOVA with post-hoc Sidak’s multiple comparisons to identify specific differences (Genotype, Time).

#### RNA isolation and sequencing of murine tendon

1M and 3M old as well as WR and CA WT and Prkaa1^Scx-Cre^ cKO mice were euthanized and Achilles tendons were immediately dissected under ribonuclease (RNase)–free conditions and flash frozen (*n* = 3/group). Tissues were mechanically pulverized in TRIzol, and total RNA was isolated using spin-columns (with on column genomic DNA digestion. RNA Integrity Number (RIN) was measured using Bioanalyzer (Agilent). This pool was subjected to 151bp paired-end sequencing according to the manufacturer’s protocol (Illumina NovaSeqXPlus). BCL Convert Conversion Software v4.0 (Illumina) was used to generate de-multiplexed Fastq files. Reads were trimmed using Cutadapt v4.8^57^ and FastQC v0.11.8 was used to ensure the quality of data^58^. Next, Fastq Screen v0.15.3 was used to screen for various types of contamination (Wingett and Andrews, 2018). Reads were then mapped to the reference genome GRCm38 (ENSEMBL), using STAR v2.7.8a^59^ and assigned count estimates to genes with RSEM v1.3.3^60^. Alignment options followed ENCODE standards for RNA-seq. Multiqc v1.20 compiled the results from several of these tools and provided a detailed and comprehensive quality control report^61^. Differential gene expression was determined from count matrices with a paired design (e.g., WT v cKO, WT WR v. CA or cKO WR v. CA) in DESeq2 in R/Bioconductor^54^. We used the Database for Annotation, Visualization, and Integrated Discovery (DAVID) to analyze biological processes and Kyoto Encyclopedia of Genes and Genomes (KEGG) pathways^55,56^.

#### Histology Preparation and Imaging

Distal hindlimbs from WT and cKO tendons (n = 3 per sex/group) were dissected, fixed in 4% paraformaldehyde (PFA), and decalcified in 14% ethylenediaminetetraacetic acid (EDTA) and processed for paraffin embedding. Tissues were sectioned at 5 μm in the sagittal plane and stained with either Hematoxylin and Eosin (H&E), Silver Nitrate, Picrosirius Red (PSR) or processed for immunohistochemistry (IHC), and cover-slipped with an acrylic mounting media. H&E, Silver Nitrate and (IHC) slides were imaged on a bright-field microscope (ECLIPSE Ni-U, Nikon) at 10x and analyzed using QuPath. PSR slides were imaged with a 10x objective on an epi-fluorescent microscope (dmi600b, Leica) and analyzed using quantitative polarized light imaging (qPLI) analysis.

#### Immunohistochemistry

For immunohistochemistry, paraffin sections were deparaffinized followed by heat-mediated antigen retrieval with an Antigen Unmasking Solution, Citrate-Based or Tris-EDTA (pH 9.0) for 2hrs at 65°C and blocking (2.5% horse serum). Slides were then incubated in primary P16-INK4A and p21 antibodies overnight at 4°C, followed by washing and incubation with appropriate secondary antibodies. ImmPACT DAB Substrate Kit, Peroxidase (HRP) was used for detection, and slides were counterstained with hematoxylin and cover-slipped with Shandon Mount. Antibody Information Table 2.

#### qPLI Analysis

The degree of linear polarization (DoLP) and angle of linear polarization (AOP) images were acquired using a polarization camera and a circular polarizing lens. The mean DoLP and standard deviation of the AOP were analyzed using Math and SciPy Stats libraries in. Data from cKO mice were compared to WT controls at 1M, 3M and 9M using a two-way ANOVA with post-hoc Sidak’s multiple comparisons to identify specific differences (Genotype, Time).

#### FIJI Analysis

Silver Nitrate slides were imaged and collected for percent lesion area analysis by FIJI (NIH, Bethesda, MD). First, the Achilles tendon region of interest area was selected using morphological features to determine total area. Next, using the polygon shape tool, the positive lesion was outlined and the area was measured. All measurements were taken three times to ensure reproducibility. Percent lesion area was calculated by dividing lesion area(s) by the whole tendon area. Data from Prkaa1^Scx-Cre^ cKO mice were compared to WT controls at 9M using an unpaired two-tailed t-test.

#### QuPath Analysis

IHC slides were imaged and collected for percent positive analysis by QuPath^62^, an open-source software for digital pathology. First, the regions of interest in Achilles tendons were selected using morphological features and areas were measured. Next, color deconvolution was performed by QuPath to digitally separate stains (Hematoxylin and DAB) within each tissue sample. Positive cell detection, highlighting DAB positive cells, was then performed to differentiate cells. Total cell detection was collected from counting hematoxylin positive nuclei. Data from Prkaa1^Scx-Cre^ cKO mice were compared to WT controls at 3M and 9M using an Ordinary two-way ANOVA with uncorrected Fisher’s LSD, with a single pooled variance to identify specific differences (Genotype, Time).

#### Nuclear Circularity

Slides were deparaffinized and stained with antifade mountant with NucBlue. Slides were imaged with widefield fluorescence on a Cytation 10 at 20x. Nuclei counts and circularity were calculated with the Gen5 cellular analysis tool. Data from Prkaa1^Scx-Cre^ cKO mice were compared to WT controls using a one way ANOVA with Tukey’s multiple comparisons test with a single pool variance.

#### Cell Isolation and Culture

Prkaa1^Scx-Cre^ cKO mice were euthanized and tails were removed. Skin was removed from tail and tendon fascicles were carefully dissected and placed in a 60mm petri dish with Dulbecco’s Modified Eagle’s Medium/Nutrient Mixture F-12 (DMEM: F12), 10 % Fetal Bobine Serum (FBS) and 1% Penicillin/Streptomycin (P/S), media was changed every other day. Tail TFs were allowed to “crawl out” for two weeks before passaging for β-Galactosidase staining and ECM arrays.

#### β-Galactosidase (Beta-Galactosidase) Staining

Prkaa1^Scx-Cre^ cKO Tail TFs were passaged into a 12w plate in DMEM: F12, 10% FBS and 1% P/S for 24h. Media was changed to DMEM: F12, 1% FBS and 1% P/S for 96h, media was changed every other day. Cells were fixed and stained per manufacturer’s instructions with the Senescence β-Galactosidase Staining Kit and imaged on an Agilent Lionheart RX.

#### ECM Arrays

Prkaa1^Scx-Cre^ cKO Tail TFs were passaged onto a per manufacturer’s instructions in DMEM: F12, 10% FBS and 1% P/S for 24h. After 24h, cells were fixed with 4% PFA and, rinsed with PBS stained with Hoechst. Slides were imaged using a Leica THUNDER and nuclei were counted using FIJI with the Stardist plugin^63^. Counts were collected and analyzed with Bayesian statistical modeling.

#### Bayesian Statistical Modeling

Nuclear Counts for the WT and Prkaa1^Scx-Cre^ cKO Tail TFs on different substrates were compared with the fit of a range of two Bayesian regression models, baseline and interaction. In the baseline model the cell counts are assumed to occur assumed to occur independently at random only. With the interaction model, the cell counts are dependent on the genotype and substrate.

### QUANTIFICATION AND STATISTICAL ANALYSIS

For graphs in figures 2,4 and 5 data are shown as mean ± standard deviation (SD). GraphPad (GP) p-value style, * p<0.05, ** p<0.01, ***p<0.001, ****p<0.0001. Statistical differences were evaluated using two-way ANOVA with Sidak correction or otherwise stated in figure legends. Graphs were produced and statistical analyses were performed using GraphPad Prism. For figure 3 data were compared with the fit of a range of two Bayesian regression models, baseline and interaction.

## Supplementary Material

Supplement 1

## Figures and Tables

**Table:** Key resources table

REAGENT or RESOURCE	SOURCE	IDENTIFIER
Antibodies		
P16-INK4A Polyclonal antibody	Proteintech	Cat# CL555–10883, RRID:AB_2923890
P21 Polyclonal antibody	Proteintech	Cat# CL594–10355, RRID:AB_2934700
ImmPRESS (Peroxidase) Polymer Anti-Rabbit IgG Reagent	Vector Labs	Cat# MP-7401–15
Chemicals, peptides, and recombinant proteins		
Harris Hematoxylin	StatLabs	Cat# HXHHEPT
Eosin Y	StatLabs	Cat# STE0157
Picro-Sirius Red Pint Stain Kit, Pint	StatLabs	Cat# KTPSRPT
Hematoxylin QS Counterstain	VectorLabs	Cat# H-304–100
Silver Nitrate	FisherScientific	Cat# BP2546100
Sodium Thiosulfate	FisherScientific	Cat# A118P-500
Formic Acid	FisherScientific	Cat# S446–500
Antigen Unmasking Solution, Citrate-Based	Vector Labs	Cat# H-3300–250
Epredia^™^ Shandon-Mount^™^	FisherScientific	Cat# 1900331
ImmPACT DAB Substrate Kit, Peroxidase (HRP)	Vector Labs	Cat# SK-415
DMEM/F12 Gibco	ThermoFisher Scientific	Cat# 11320033
RNAlater	ThermoFisher Scientific	Cat # AM7020
Critical commercial assays		
PureLink RNA mini kit	ThermoFisher	Cat# 12183018A
RNase-free DNase	Qiagen	Cat# 79254
SuperScript IV VILO Master Mix	ThermoFisher	Cat# 11756050
Power SYBR Green PCR Master Mix	ThermoFisher	Cat# 4367659
Senescence Beta-Galactosidase Staining Kit	Cell Signaling	Cat# 9860S
RNeasy Mini Fibrous Tissue Kit	Qiagen	Cat # 74704
NEBNext Ultra II RNA Library Preparation for Illumina Kit	New England Bio Labs	Cat # E7770S
Dual Index Primers	New England Bio Labs	Cat # E3392S
Deposited data		
Human Tendinopathy Bulk RNA Sequencing	This Paper; Gene Expression Omnibus	
Prkaa1^Scx-Cre^ cKO Bulk RNA dataset	This Paper; Gene Expression Omnibus	
Experimental models: Organisms/strains		
C57BL/6-*AMPKa1*^*flox/flox*^	The Jackson Laboratory	JAX:014141
C57BL/6-Tg(Scx-GFP/cre)1Stzr	Gifted from Ronen Schweitzer	MGI ID: 5317398
Software and algorithms		
SciPy Stats library	Python	
QuPath 0.5.1	QuPath	https://qupath.github.io
ImageJ	NIH	https://imagej.nih.gov/ij/
Prism v10	GraphPad	
MetaShape	Agisoft	
Adobe Illustrator	Adobe	https://adobe.com
RStudio	The R Foundation	https://cran.r-project.org/
R	The R Foundation	https://cran.r-project.org/
BioTek Gen5 Software for Imaging & Microscopy	Agilent	
MATLAB	Mathworks	
Database for Annotation, Visualization, and Integrated Discovery (DAVID)	NIH	https://davidbioinformatics.nih.gov/summary.jsp; doi:10.1093/nar/gkac194; DOI: 10.1038/nprot.2008.211
BCL Convert Conversion Software v4.0	Illumina	
Cutadapt v4.8		DOI:10.14806/ei.17.1.200.
FastQC v0.11.8	Babraham Bioinformatics	https://bioinformatics.babraham.ac.uk/projects/fastqc/
Fastq Screen v0.15.3	Babraham Bioinformatics	https://www.bioinformatics.babraham.ac.uk/projects/fastq_screen/
GRCm38	National Library of Medicine	https://api.ncbi.nlm.nih.gov/datasets/v2/genome/accession/GCF_000001635.20/download?include_annotation_type=GENOME_FASTA&include_annotation_type=GENOME_GFF&include_annotation_type=RNA_FASTA&include_annotation_type=CDS_FASTA&include_annotation_type=PROT_FASTA&include_annotation_type=SEQUENCE_REPORT&hydrated=FULLY_HYDRATED
STAR v2.7.8a		https://doi.org/10.1093/bioinformatics/bts635
RSEM v1.3.3		https://doi.org/10.1186/1471-2105-12-323
Multiqc v1.20	Seqera	https://docs.seqera.io/multiqc/getting_started/installation
Other		
Kiralux Polarization Camera, 5 MP Monochrome CMOS Sensor, USB 3.0 Interface	ThorLabs	Cat# CS505MUP1
V500C	Biomomentum	
NovaSeqXPlus	Illumina	
ECM Select^®^ Array Kit Ultra-36	Advanced Biomatrix	Cat# 5170
Eclipse Ni-U	Nikon	
THUNDER	Leica	
Cytation 10	Agilent	
50mm Dia. Left-Handed Plastic Circular Polarizer	Edmund Optics	Cat# 88–099
Thin Film Grip	Imada	
MA160	Biomomentum	
Ball Mill Homogenizer	Qiagen	
Qubit fluorometer	Life Technologies	
Bioanalyzer	Agilent	
Illumina NextSeq500 Instrument	Illumina	Cat # SY-415–1002
